# The Effects of Teacher Feedback and Automated Feedback on Cognitive and Psychological Aspects of Foreign Language Writing: A Mixed-Methods Research

**DOI:** 10.3389/fpsyg.2022.909802

**Published:** 2022-07-28

**Authors:** Zehua Wang, Feifei Han

**Affiliations:** ^1^School of Foreign Languages, Shaanxi Xueqian Normal University, Xi’an, China; ^2^Institute for Learning Sciences and Teacher Education, Australian Catholic University, Sydney, QLD, Australia

**Keywords:** teacher feedback, automated feedback, foreign language writing, revision quality, writing proficiency development, perceived usefulness, perceived ease of use, perceptions of the feedback

## Abstract

Feedback plays a crucial role in the writing processes. However, in the literature on foreign language (FL) writing, there is a dearth of studies that compare the effects of teacher feedback and automated feedback on both cognitive and psychological aspects of FL writing. To fill this gap, the current study compared the effects of teacher feedback and automated feedback on both revision quality and writing proficiency development (i.e., the cognitive aspects), and perceived usefulness and perceived ease of use of the feedback (i.e., the psychological aspects) in English writing among English learners as an FL (EFLs) in China. It also investigated students’ perceptions of the strengths and weaknesses of the two types of feedback. The study adopted a mixed-methods design. The quantitative method collected the data through (1) a pre-test and a post-test, which measured the participants’ English writing proficiency development; (2) a writing task, which received either teacher feedback or automated feedback; and (3) a close-ended questionnaire, which examined students’ perceived usefulness and perceived ease of use of the feedback. The qualitative method collected the data through an open-ended questionnaire, which examined the participants’ perceptions of the strengths and weaknesses of teacher feedback or automated feedback depending on the type of feedback they received. Chinese university EFLs in two English classes (*n* = 35 in each class) taught by the same English teacher participated in the study: one class received teacher feedback while the other received automated feedback using Pigaiwang. While the students in the two classes did not differ significantly on the pre-test of students’ writing proficiency, students who received teacher feedback scored significantly higher on revision than those who received automated feedback. Students in the teacher feedback class also had significantly higher ratings on perceived usefulness and perceived ease of use of the feedback than those in the automated feedback class. However, students in the automated feedback class obtained significantly higher scores on the post-test of the writing proficiency. The qualitative results identified three themes of strengths and two themes of weaknesses for the teacher feedback and the automated feedback, respectively. The results suggest that while teacher feedback has a more positive effect on the psychological aspect of FL writing, automated feedback may be more effective in developing FL writing proficiency in the long run.

## Introduction

In early 2020, the COVID-19 pandemic spread rapidly worldwide, which brought great challenges to all walks of life, including educational institutions. In order to ensure the normal progress of learning and at the same time to prevent the spread of COVID-19, institutions across countries were required to redeploy more learning and teaching activities to virtual learning spaces in order to maintain physical distancing. As a result, the vast numbers of face-to-face courses have been delivered either as blended courses or as purely online courses (Tang et al., 2021). Under such circumstances, technology-supported learning, particularly freely accessible web-based tools of high quality have been playing an important role in the learning and teaching processes. In foreign language (FL) writing, one such useful tool is web-based automated writing feedback systems.

A web-based automated writing feedback system is also known as online automated writing feedback, which is a type of an online platform that can be flexibly and easily accessed by students and provide immediate feedback to them (Warschauer and Grimes, 2008). The online automated feedback can also enable teachers to identify an individual student’s level of writing ability in relation to the whole class when all students’ essays are entered into the system (Bai and Hu, 2017). Because of these benefits, online automated feedback platforms have been increasingly adopted by English teachers around the world to fully or partially replace teacher feedback in English writing classes (Warschauer and Ware, 2006).

The functions of automated feedback have been recognized as both an assessment and a learning tool. Research on the effectiveness of automated feedback as an assessment tool has demonstrated that automated feedback is advantageous for its ability to significantly reduce logistics for marking a large number of written scripts and to evaluate writing more objectively than using human raters (Shermis and Burstein, 2003; Wilson et al., 2014). These merits make automated feedback particularly favorable for scoring written scripts of test-takers in large-scale standardized tests (Ramineni, 2013). As a learning tool, automated feedback can provide feedback on learners’ writing in various aspects, including mechanics, vocabulary, grammar, and content and organization, which not only can assist learners in improving the quality of writing products, but may also help them acquire additional linguistic knowledge, such as learning new words by reading the synonyms offered by automated feedback (Wilson and Czik, 2016).

However, there is little research comparing the effects of teacher feedback and automated feedback on both FL learners’ revision quality and their writing proficiency development (the cognitive aspects of FL writing) in a single study. Moreover, from a psychological perspective, it is also important to know how learners perceive the usefulness and ease of use of automated feedback compared to teacher feedback, as operating automated feedback requires a certain level of knowledge of using computers. From a teaching point of view, in order to help teachers make informed decisions as to how much the two types of feedback should be used in the FL writing classes, students’ perceptions of the strengths and weaknesses (the psychological aspects of FL writing) of the two types of feedback also should be understood. To address these research gaps, the current study compares the effects of teacher feedback and automated feedback on both the cognitive and psychological aspects of FL writing among learners of English as an FL (EFL learners) in China. The following sections review relevant literature on both teacher feedback and automated feedback.

## Literature Review

### Feedback in Foreign Language Writing

Revising is an important part of the writing process, especially when writing in an FL. FL learners who routinely revise inadequacies in their texts after receiving feedback tend to develop better writing skills than those who do not (Ferris and Roberts, 2001; Bitchener, 2008). To help FL learners achieve a desirable revision quality in writing, providing various types of feedback about their writing is of great importance. There are a variety of types of feedback, including corrective, non-corrective, direct, indirect, local, and global (Luo and Liu, 2017). The feedback can also focus on different features in the writing, such as mechanics, grammatical errors, vocabulary and collocations, and content and structure of the writing (Lee, 2008; Boubekeur, 2015). Researchers suggest that the good practice of writing feedback should cover both form and content in writing (Biber et al., 2011).

One major issue that has been consistently addressed in FL writing is how to provide effective feedback (Lee, 2017). Researchers and educators have proposed both unfocused and selective approaches to provide feedback; however, the two approaches tend to target learners with different levels of FL proficiency (Ferris et al., 2013). In the unfocused approach, teachers give comprehensive feedback to students, responding to every single error, whereas in the selective approach, teachers only target a selected type of error in their feedback (Lee, 2013). Hence, the unfocused approach is more suitable for advanced learners, as their writings tend not to have a large number of errors. On the other hand, the selective approach is more appropriate for less proficient learners, because selective feedback can avoid overwhelming learners, at the same time enable them to notice the focused types of errors (Sheen, 2007; Bitchener, 2008; Bitchener and Ferris, 2012). As to whether direct or indirect feedback is more effective for FL learners, there is no conclusive evidence. Some researchers believe that indirect feedback provides learners opportunities to use their existing knowledge to correct the errors themselves, which not only can engage learners in the revising processes but tends to be more effective to promote accuracy in writing in the long term (e.g., Ferris, 2006; Bitchener and Ferris, 2012). More recent studies, however, reported that it seemed to be more effective to provide direct corrective feedback involving metalinguistic explanations, through which learners’ cognitive engagement could also be enhanced (Bitchener and Knoch, 2008, 2009, 2010; Esfandiar et al., 2014).

### Teacher Feedback on the Revision Quality and the Writing Proficiency Development

A large number of studies have examined the effect of teacher feedback on FL learners’ revision, which demonstrate that different types of teacher feedback have different effects on learners’ revision (Hyland and Hyland, 2006). Research has reported that teachers’ positive comments and encouraging language could build students’ confidence, which is considered important in the revising processes (Ferris, 1997). Studies have also suggested that in order for teacher feedback to be effective in the revising process, direct, specific, and content-related feedback should be given (Ferris, 2011). Of the three types of feedback, namely advice, criticism, and praise, research has shown that the advice type of feedback is more likely to prompt students to revise (Silver and Lee, 2007).

Compared to the research on the effect of teacher feedback on FL learners’ revision, fewer studies have investigated the effect of teacher feedback on FL learners’ writing proficiency development. The effect of teacher feedback on learners’ writing proficiency development may not be as effective as on the revision quality, as learners may simply directly copy teacher corrective feedback without understanding the errors (Hyland, 1998; Lee, 2007; Zhao, 2010). As a result, they will still make the same mistake in their subsequent writings. Zhao (2010) has suggested that teacher feedback “that is used/copied but ununderstood may help to improve writing quality but does not necessarily contribute to the development of learners’ long-term writing proficiency” (p. 4). Seeing these problems, researchers have proposed that the examination of the effects of teacher feedback should also be examined in subsequent instances of writings beyond just the revised drafts of the same text (Ruegg, 2015). Thus, it is necessary to investigate the effects of teacher feedback on students’ revision and their writing proficiency development simultaneously.

### Students’ Perceptions of Teacher Feedback

In general, FL writers attach great importance to teacher feedback, as they believe that teachers are more authoritative in giving writing feedback than their peers (Ferris, 1995; Hyland and Hyland, 2006; Biber et al., 2011). FL learners believe that teacher feedback not only helps them avoid making similar mistakes in their subsequent writing (Chandler, 2003), but also strengthens their confidence and motivation in FL writing, particularly when teachers use positive language in the feedback (Weaver, 2006).

However, teacher feedback focusing on different aspects of writing is perceived differently by learners. Some FL writers perceive that the comments on contents and structures are the most important and useful feedback (Semke, 1984; Zamel, 1985; Zhan, 2016), whereas others value feedback on form over content (Saito, 1994; Hedgcock and Lefkowitz, 1996; Ashwell, 2000; Lee, 2005). Some learners even expect to receive feedback on all aspects of their writing, including language problems, contents, and organizational structure (Radecki and Swales, 1988; Ferris, 1995; Lee, 2005). Learners may favor different types of feedback based on different reasons. Some FL writers prefer indirect feedback on the basis that the indirect type gives them more agency to actively participate in the revision processes (Hyland, 2001). In contrast, other learners welcome detailed and personalized feedback with clear explanations of the errors (Ferguson, 2011; Dawson et al., 2019).

The FL learners do not always hold positive perceptions toward teacher feedback. Some students mentioned that they either did not understand teacher feedback or they found teachers’ language in the feedback was ambiguous, hence they had to ignore these comments in the revising processes (Ferris, 1997; Conrad and Goldstein, 1999; Rollinson, 2005; Kim, 2015). These problems were particularly prominent among students with low self-efficacy in FL writing and with poor writing proficiency (Lee, 2008). While past studies have investigated students’ perceptions of teacher feedback, relatively little research has compared students’ perceptions of the strengths and weaknesses of teacher feedback and automated feedback.

### Automated Feedback on the Revision Quality and the Writing Proficiency Development

With the development of educational technology, automated feedback has been increasingly applied in English writing evaluation and instruction (Chen and Cheng, 2008; Warschauer and Grimes, 2008; Grimes and Warschauer, 2010). The initial aim of the development of automated feedback was on scoring a large number of essays in standardized writing assessments (Page, 2003). In recent years, automated feedback has been also employed in FL writing classrooms to provide timely feedback in classes with large enrolments (Stevenson and Phakiti, 2014; Liao, 2016; Wilson and Andrada, 2016). The apparent strength of automated feedback, especially web-based automated feedback, lies in its efficiency and flexibility, as it can identify errors and provide immediate feedback merely by a click on the web page (Chen and Cheng, 2008; Cotos, 2011). It is particularly effective to provide corrective feedback on the aspects of mechanics and grammatical errors (Wilson et al., 2014; Li et al., 2015). Bai and Hu (2017) reported that the correction rates of automated feedback were 57 and 42% for grammatical and collocation errors, respectively. Others found that the success rate of error corrections could be as high as 70% (Chapelle et al., 2015; Liao, 2016). Moreover, automated feedback also has the potential to reduce the burden for English teachers in terms of managing, storing, and marking FL learners’ writing samples (Manap et al., 2019).

Despite these benefits, automated feedback has been criticized for its low-quality feedback on the content and organization of the writing (Warschauer and Grimes, 2008; Wang, 2013). For instance, some popular automated feedback systems, such as *Criterion* and *My Access!*, predominantly focus on detecting language errors (Dikli, 2010), but are limited in identifying high-level problems, such as content and logic (Deane, 2013). The predominant foci of the mechanical and linguistic features generated by automated feedback may mislead FL learners to think that FL writing practice is all about language aspects, neglecting the content and rhetoric aspects of the writing (Cheville, 2004). Another concern for using automated feedback is that it requires learners to have some levels of learning autonomy so that they can sustainably interact with the machine (Warschauer and Ware, 2006). Thus, automated feedback may not be suitable for younger FL learners due to their lack of learning autonomy (Lee, 2017).

### Students’ Perceptions of Automated Feedback

As to students’ perceptions of automated feedback, the majority of the existing research has explored students’ perceptions of automated feedback in the context of first language writing (Calvo and Ellis, 2010; Grimes and Warschauer, 2010). In the FL writing, the limited research has produced mixed results (Chen and Cheng, 2008; Calvo and Ellis, 2010; Dikli and Bleyle, 2014; Bai and Hu, 2017). While some researchers have reported that students hold negative perceptions of automated feedback as they believe that automated feedback does not provide sufficient information on the contents of the writing (Chen and Cheng, 2008), others have positive attitudes due to the flexibility in accessing automated feedback (Fang, 2010). Students also have different perceptions as to the automated feedback on different aspects of FL writing. While most students have positive perceptions of the feedback mechanics and grammar provided by the automated feedback, they showed some concerns about the reliability of the feedback on collocations (Bai and Hu, 2017).

Studies investigating students’ perceived usefulness and ease of use of automated feedback seemed scarce. Perceived usefulness refers to the degree to which a user perceives that using a particular technology system would enhance his/her performance (Davis, 1989), whereas perceived ease of use is defined as the degree to which a user expects that using a particular technology system is free of effort (Davis, 1989; Venkatesh and Davis, 2000). These two constructs are the most important constructs in the Technology Acceptance Model (Davis, 1989; Neo et al., 2015), and have been widely researched in users’ experience of using e-learning and technology systems (Zyad, 2016). As the web-based automated feedback system is also an e-learning system, examination of FL writers’ perceived usefulness and ease of use of the automated feedback system is important.

### Comparing Teacher Feedback and Automated Feedback in Foreign Language Writing

To date, only a small number of studies have compared the effects of teacher feedback and automated feedback on FL writing (Warden, 2000; Wang and Wang, 2012; Dikli and Bleyle, 2014; Wilson and Czik, 2016; Link et al., 2020). However, these studies suffer from some design issues. Warden reported a better effect of automated feedback on reducing learners’ error rates than teacher feedback. In his study, the feedback from automated feedback was on specific errors, whereas teacher feedback was general comments. Similarly, the participant in the teacher feedback condition in Wang and Wang’s study also only received the global comments on his writing, whereas the participant in the automated feedback condition received the specific comments on grammar, spelling, and collocations. Moreover, this study only had two participants, which severely limited the generalizability of the findings. In Link et al.’s (2020) and Wilson and Czik’s (2016) study, students in the automated feedback conditions received a combination of automated feedback and teacher feedback. Hence, the comparison was not purely between teacher feedback and automated feedback. To address these methodological issues, the current study will (1) have pure teacher feedback and a pure automated feedback condition, and (2) require the teacher to provide feedback by covering all the aspects in writing, which are covered in automated feedback.

### The Current Study and Research Questions

The literature review shows that there is a lack of research comparing the effects of teacher feedback and automated feedback on both cognitive and psychological aspects of FL writing. To fill this gap, the current study compared the effects of teacher feedback and automated feedback on both revision quality and writing proficiency development (i.e., the cognitive aspects), and perceived usefulness and perceived ease of use of the feedback (i.e., the psychological aspects) in English writing among English learners as an FL (EFLs) in China. It also investigated students’ perceptions of the strengths and weaknesses of the two types of feedback. The current study sought to answer the following three research questions:

(1) To what extent do revision quality and writing proficiency development differ between Chinese EFLs who receive teacher feedback and automate feedback?
(2) To what extent do perceived usefulness and ease of use differ between Chinese EFLs who receive teaching feedback and automated feedback?
(3) What are the strengths and drawbacks of teacher feedback and automated feedback perceived by Chinese EFLs?

## Method

### Research Design

The study adopted a mixed-methods design: the quantitative method provided the answers to the first and the second research questions, whereas the qualitative method provided the answer to the third research question. For the quantitative method, we conducted a quasi-experiment as it was not possible to randomly assign the participants into two groups due to the university’s policy. Hence, we designed the quasi-experiment on the basis of the two intact classes: one class received teacher feedback and the other received web-based automated feedback on their English essay drafts. The quantitative part also collected students’ responses to perceived usefulness and ease of use of either teacher feedback or automated feedback, depending on which one they received, through a Likert-scale questionnaire. The qualitative method obtained students’ perceptions of the strengths and drawbacks of teacher feedback and automated feedback through an open-ended questionnaire. In the following sections, details regarding the participants, instruments, data collection, and analysis methods are explained.

### Participants

A total of 70 Chinese freshmen, who majored in English Education in Early Childhood participated in the study. Among them, 67 were women and only three were men. The uneven gender distribution was largely attributable to the fact that the major of Early Childhood Education generally attracts female students in China. The 70 students attended two English classes taught by the same English teacher, with each class having 35 students. The participants were aged between 18 and 21 years, with an average of 19.5 years. All the participants had studied English as a compulsory subject for 9 years, from grade three in primary school to completion of high school. At the time of the data collection, students had just commenced their university studies, hence they did not have opportunities to take part in any national examinations for college students. Therefore, we gathered students’ English scores from the National College Entrance Examinations as an indicator of their English proficiency. The total score of the National College Entrance English Examination is 150. Of the 70 participants, 34 had scores ranging from 85 to 100, 28 ranging from 100 to 110, and 8 ranging from 110 to 120. Therefore, their English proficiency could be placed at the lower intermediate to intermediate levels.

### Instruments

#### The Writing Tasks

Three writing tasks were used in the study. The first writing task served as a pre-test of participants’ English writing proficiency in order to examine if students in the two English classes had similar English writing proficiency before the quasi-experiment. As the participants had just commenced their university life, we used a writing task titled “My first day at the university,” which was considered appropriate and relevant to students’ life experiences. A one-way ANOVA was conducted to examine if the students in the two feedback conditions had the same initial writing proficiency. Levene’s test found that the assumption of homogeneity of variances was met [*F*(1,68) = 0.76, *p* = 0.38]. The results showed that there was no significant difference [*F*(1, 68) = 0.31, *p* = 0.58, η^2^ = 0.13] in the pre-test of English writing proficiency between students receiving teacher feedback (*M* = 66.66, SD = 7.73) and those receiving automated feedback (*M* = 67.63, SD = 6.75).

The second writing task, which was titled “The most impressive classmate in my university,” was used to provide feedback to students for them to revise. The third writing task was called “The most successful thing I have done,” which was used to test students’ writing proficiency after the quasi-experiment. All three writing tasks required students to produce an approximate 150-word English text following a structure of three compulsory parts, namely, an introduction, a body text, and a conclusion. As past research suggests that text type can affect FL writers’ writing performance (Li, 2014), we therefore used a single text type, that is, narratives for all three tasks. We purposefully chose to use narratives rather than expositions or other text types for the writing tasks, because the participants were familiar with this text type. The topics of the three writing tasks came from the category of the daily practice of narratives for English major students in China in the bank of the web-based automated feedback used in the study in order to ensure that the three writing tasks had similar difficulty levels.

#### The Writing Feedback

The online automated feedback used in this study was called “Pigaiwang,” whose word-to-word English translation is “Marking Website” (see http://www.pigai.org/ for an example of the interface of the Pigaiwang). Entering the market in 2011, this platform has registered as a patent in China (ZL2012 10049982.4). It is both a corpus-based and a cloud-computing-based online service for automated evaluation and feedback of English writing by Chinese EFLs. The reliability and the validity of the Pigaiwang for English essay scoring were established by calibrating with a large corpus of human-scored English essays (Hu, 2015; Yang and Dai, 2015). It has been reported that the correlation between essay scores in the Pigaiwang and human raters was high and satisfying (He, 2013; Wang, 2016). With years of development, the platform has evolved into the most popular and most widely subscribed web-based automated feedback platform in China, with more than 20 million registered users by early 2018.

The Pigaiwang provides four main functions for learners:

• Scoring the essay: this function computes a score immediately upon the submission of an essay to indicate the quality of English writing. Each essay is scored on the four dimensions of vocabulary, grammar, structure and organization, and content and relevance, each of which is scored by comparing the quality of the submitted essays with a large human-scored essay corpus. The possible writing scores range from 0 to 100. In addition to the scores, when students are registered under one class, the system will also generate a rank of their writing quality relative to the whole class performance.
• Providing immediate holistic feedback: this function uses both bar graphs and comments to demonstrate the strengths of an essay in terms of vocabulary, grammar, structure and organization, and content and relevance.
• Providing corrective feedback at the sentence level: this function provides diagnostic comments by pointing out the errors in mechanics (e.g., spelling, punctuations, and capitalization), vocabulary (e.g., word choice and collocation), grammar, and content and relevance. It also gives suggestions for revision at the level of individual sentences and recommendations for collocations. The recommended collocations are listed in ranks according to the frequency of the appearance in corpora.
• Listing suggestions for synonyms at the level of a word: this function offers the writers multiple synonyms in order to enhance the vocabulary diversity of the writing. For each synonym, it also supplies hyperlinks for further information on the meaning of the synonyms and detailed explanations as to the differences between the synonyms and the word appearing in the submitted texts.

The English teacher’s feedback matched the format of the feedback generated in the Pigaiwang. Table 1 provides some examples of the teacher feedback by types of errors.

**TABLE 1:** Examples of teacher feedback by types of errors.

Types of errors		Errors	Teacher feedback
Mechanics	Spelling	There are six girls in my dormatory.	a spelling error
	Punctuations	In a word ^ my college life is busy.	an issue with punctuation
	Capitalization	All My classmates love playing table tennis.	capitalization problem
Vocabulary	Collocation	She always looks for me as a big sister.	a problem with collocation
	Word choice	Reading is a good way to increase my English proficiency.	a problem with word choice: “improve” should be used.
Grammar		Receive positive words from my friends is very important for me.	A noun is needed.

*The underlined sections indicate errors in students’ writing.*

#### The Likert-Scale Questionnaire on Perceived Usefulness and Ease of Use of the Feedback

To measure students’ perceived usefulness and ease of use of the feedback they received, two 5-point Likert scales were adapted from the existing scales. The items were adapted from Venkatesh et al. (2003) and Venkatesh and Bala (2008), which were originally developed and reported by Davis (1989) and Davis et al. (1989). The wording of the scales used for students who received teacher feedback and automated feedback was exactly the same, except for the words “teacher feedback” and “automated feedback.” The perceived usefulness scale had four items, and its reliability was 0.68 for teacher feedback and 0.67 for automated feedback. The perceived ease of use scale had three items, and its reliability was 0.69 for both teacher feedback and automated feedback.

#### The Open-Ended Questionnaire on the Strengths and Weaknesses of the Feedback

The open-ended questionnaire asked students to list three aspects of both strengths and weaknesses of either teacher feedback or automated feedback depending on which one they received.

### Ethics Consideration

Prior to the study, the students in the two classes were informed about the purposes of the study and were invited to participate in the study voluntarily. Before the data collection, an ethical application was submitted to the ethics committee of the School of Foreign Languages, Shaanxi Xueqian Normal University. The committee evaluated the nature of the study and believed that the study would be a component of classroom teaching. Hence, the participants were not required to sign a written consent form. However, all the participants needed to agree verbally for the voluntary participation. The ethics committee recorded the participants’ verbal consent.

### Procedure of the Data Collection

The research was conducted in four English sessions. In the first English session, participants in the two classes completed the first writing task. The scores were used to represent students’ initial English writing proficiency. In the second English session, both groups completed the second writing task. The students in the automated feedback class submitted their essays in the Pigaiwang, whereas students in the teacher feedback class submitted their essays to the English teacher. In the third English class, the students were instructed to revise their essays using either teacher feedback or automated feedback. The scores of the revised essays were used to represent the revision quality. In the fourth English session, they were given the third writing task. The scores of the third writing task were used to assess students’ post-English writing proficiency. Upon completion of the third writing task, they were also given both the Likert-scale questionnaire as well as the open-ended questionnaire to fill. The essays for the first writing task (pre-test of English writing proficiency), the revised essays for the second writing task, and the essays for the third writing task (post-test of English writing proficiency) were scored in the Pigaiwang to prevent marking bias of the human raters. The procedure of the data collection is summarized in Table 2.

**TABLE 2:** Summary of the data collection procedure.

Two classes	1st English session	2nd English session	3rd English session	4th English session
Teacher feedback	Students completed the first writing task (pre-test of English writing proficiency).	Students completed the second writing task and submitted their essays to the English teacher.	Students revised their essays using the teacher feedback. The revision was scored by Pigaiwang.	Students completed the third writing task (post-test of English writing proficiency).
Automated feedback	Students completed the first writing task (pre-test of English writing proficiency).	Students completed the second writing task and submitted their essays in the Pigaiwang.	Students revised their essays using the feedback from Pigaiwang. The revision was scored by Pigaiwang.	Students completed the third writing task (post-test of English writing proficiency).

### Data Analysis

To answer the first research question, comparing students’ revision quality and post-test of English writing proficiency, a mixed-design 2 (within-subjects factor: revision quality and post-test of writing proficiency) × 2 (between-subjects factor: teacher feedback vs. automated feedback) ANOVA was conducted. To answer the second research question, comparing students’ perceived usefulness and ease of use of the feedback, a MANOVA was used. To examine students’ perceptions of the strengths and weaknesses of the two types of feedback, a thematic analysis of the students’ responses to the open-ended questionnaire was applied.

## Results

### Comparison of Revision Quality and Post-test of Writing Proficiency Between Students Receiving Teacher Feedback and Automated Feedback

**FIGURE 1:**
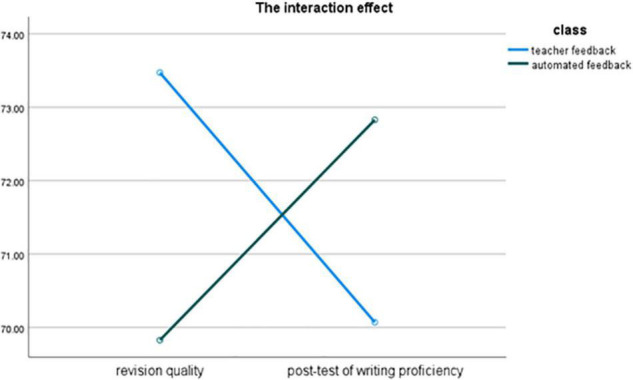
The interaction effect between writing occasion and feedback type.

As the revision quality and the post-test of writing proficiency used two different writing tasks, the result of the within-subjects effect of the 2 × 2 mixed ANOVA was not relevant to the current study. The result of the interaction effect between writing occasion and feedback type was significant [*F*(1, 68) = 10.93, *p* < 0.01, η^2^ = 0.19], suggesting that the patterns of students’ scores on revision quality and the post-test of writing proficiency were different by feedback type (see Figure 1).

Separate one-way ANOVAs were conducted for revision quality and post-test of writing proficiency. For revision quality, Levene’s test showed that the assumption of homogeneity of variances was met [*F*(1,68) = 1.15, *p* = 0.29]. The one-way ANOVA demonstrated that [*F*(1, 68) = 8.76, *p* < 0.01, η^2^ = 0.11] students who received teacher feedback (*M* = 73.47, SD = 5.59) scored significantly higher than those who received automated feedback (*M* = 69.83, SD = 4.67). For the post-test of writing proficiency, Levene’s test also confirmed that the assumption of homogeneity of variances was not violated [*F*(1,68) = 0.24, *p* = 0.63]. The results of the one-way ANOVA showed that the pattern was reversed [*F*(1, 68) = 4.02, *p* < 0.05, η^2^ = 0.06]. Students in the automated feedback class (*M* = 72.83, SD = 5.54) received significantly higher scores than their peers in the teacher feedback class (*M* = 70.07, SD = 5.59).

As the students in the two classes did not differ in terms of their pre-test of English writing proficiency, the significantly better revision scores of the students who received teacher feedback suggested that teacher feedback was more effective in helping students revise their essays. In contrast, the significantly higher post-writing proficiency of the students who received automated feedback indicated that automated feedback might be more effective in developing FL learners’ writing proficiency.

### Comparison of Perceived Usefulness and Perceived Ease of Use Between Students Receiving Teacher Feedback and Automated Feedback

The results of the MANOVA found that the feedback type was significant [*F*(2, 67) = 9.34, *p* < 0.01; Wilk’s Λ = 0.78, partial η^2^ = 0.22]. Levene’s tests confirmed that the assumption of homogeneity of variances was met for both perceived usefulness scale [*F*(1,68) = 0.29, *p* = 0.59] and perceived ease of use scale [*F*(1,68) = 0.20, *p* = 0.65]. Univariate tests showed that students in the two different feedback conditions differed significantly on both perceived usefulness [*F*(1, 68) = 18.42, *p* < 0.01, η^2^ = 0.21] and perceived ease [*F*(1, 68) = 4.19, *p* < 0.05, η^2^ = 0.06] scales. Specifically, students who received teacher feedback (*M* = 3.97, SD = 0.44) had significantly higher ratings on the perceived usefulness scale than their peers who received automated feedback (*M* = 3.46, SD = 0.54). Students who received teacher feedback (*M* = 4.05, SD = 0.58) also had significantly higher ratings on the perceived ease of use scale than their counterparts who received automated feedback (*M* = 3.75, SD = 0.62).

### Perceptions of the Strengths and Weaknesses of Teacher Feedback and Automated Feedback

The participants had mixed feelings toward both teacher feedback and automated feedback. Table 3 summarizes the themes of the strengths and weaknesses of both types of feedback and the frequency of each theme. It should be noted that the total frequency of all the themes did not equate to the number of students, as some students only wrote about the strengths or the weaknesses, while some noted down more than one response to the strengths and the weaknesses.

**TABLE 3:** Themes of the strengths and weaknesses of teacher feedback and automated feedback.

	Strengths	Frequency	Weaknesses	Frequency
Teacher feedback	a balanced comments on both the positive and negative aspects of students’ writing	20	lacking detailed explanations	4
	encouraging words	17	too many comments	4
	clarity and easiness of the language use	12		
Automated feedback	the ability to identify errors	32	ambiguous and incomprehensible	21
	the capacity to suggest for synonyms	16	too much emphasis on mechanical problems, such as punctuation and capitalization problems	9
	the function to rank essays to allow students to know their ranks in relation to their classmates	11		

The most frequently mentioned (mentioned by 20 students) strength of teacher feedback was that teacher feedback had balanced comments on both the positive and negative aspects of the students’ writing. For instance, a student commented: “I like teacher feedback because it not only pointed out the problems and mistakes in my writing, but also included good comments on my essay. To me, this is really important, because these good words made me more confident about my English writing proficiency and encouraged me to make efforts to revise my essay.” The second frequent strength of teacher feedback was the encouraging words used by the English teacher (mentioned by 17 students). These students believed that these encouraging words in teacher feedback enhanced their motivation and fostered their enthusiasm for English writing in the future, including the subsequent revisions. The third frequently mentioned strength of teacher feedback (mentioned by 12 students) was clarity and easiness of the language use, which, according to students, was easy to comprehend and hence can improve the efficiency of their revising process.

When looking at the positive comments on automated feedback, we found that the participants predominantly focused on automated feedback’s ability to identify errors (mentioned by 32 students). An example response was: “One of the positive aspects of automated feedback is that it identifies the errors in my writing, by correcting these errors, my revised essay would be improved.” This answer seems to suggest that the majority of students receiving automated feedback used such feedback as a mistake identification tool for them to fix errors in their English writing. The second most frequently mentioned strength (mentioned by 16 students) of automated feedback was that it provided suggestions for synonyms and detailed explanations of the differences between the synonyms. For instance, a student made such a comment: “Automated feedback is particularly good at offering multiple synonyms for me to choose. So I can use different words in my writing rather than always repeat the same word. I feel that this kind of feedback generated by Pigaiwang can enlarge my vocabulary size.” The next most frequently mentioned strength (mentioned by 11 students) of automated feedback was its ability to provide a rank, which allowed the students to know their writing ability in relation to their fellow students.

In terms of the shortcomings, only a few students mentioned issues in teacher feedback. Four students believed that the feedback received from the English teacher sometimes lacked detailed explanations, as the teacher simply underlined the sentences or highlighted the words. Four students commented that there were too many comments from the English teacher. In contrast, as many as 21 students pointed out that the comments generated by automated feedback were not always straightforward and comprehensible, which created barriers for them to revise their essays properly. Nine students mentioned that automated feedback emphasized too much on mechanical problems, such as punctuation and capitalization problems.

## Discussion and Conclusion

### The Effects of Teacher Feedback and Automated Feedback on the Cognitive Aspects of Foreign Language Writing (Revision Quality and Writing Proficiency Development)

In terms of the effects of teacher feedback and automated feedback on the cognitive aspects of FL writing, we found that students who received teacher feedback scored significantly higher on revision quality than those who received automated feedback, whereas students in the automated feedback class showed better performance on the post-test of their writing proficiency. The different effects of the two types of feedback on revision quality and on writing proficiency development suggest that teacher feedback and automated feedback may play different roles in helping FL writers revise and enhance their writing proficiency.

One of the possible reasons for the better effect of teacher feedback on the revision quality could be the low level of the English writing proficiency of our participants. Research has shown that students with low writing proficiency tend to overly rely on teacher feedback in the revision process (Zhang, 2020). The limited effect of teacher feedback on developing participants’ writing proficiency could be that our participants might directly copy teacher feedback in the revision without knowing their problems, as shown in previous research (Lee, 2007; Zhao, 2010). Without knowing the sources of errors, participants would make the same mistakes again in their subsequent writing.

The reason for the better effect of automated feedback on developing our participants’ writing proficiency may lie in its capacity to offer suggestions for synonyms, which may have enlarged our participants’ vocabulary in the long run. Past research has reported that FL learners’ receptive and productive vocabulary size is strongly associated with their writing proficiency, as a large vocabulary size allows students to express richer ideas in writing (Staehr, 2008; Shi and Qian, 2012; Lee, 2014; Lin, 2015; Miralpeix and Muñoz, 2018). For instance, Lee found that Korean university EFL learners’ productive vocabulary size had significant effects on multiple aspects of their writing, including both content and language aspects. Similarly, among 67 Hong Kong university EFL learners, Lin reported that students’ performance on the two vocabulary tests, namely Vocabulary Levels Test and Word Associates Test, could explain a quarter of their English writing performance. As we did not measure the vocabulary change of our participants in the automated feedback class, whether students who received automated feedback performed better on the post-test of the writing proficiency was related to their increased vocabulary needs further verification.

### The Effects of Teacher Feedback and Automated Feedback on the Psychological Aspects of Foreign Language Writing (Students’ Perceived Usefulness and Ease of Use, Their Perceptions of the Strengths and Weaknesses of the Feedback)

In terms of the effects of the two types of feedback on the psychological aspects of FL writing, we found that students in the teacher feedback class had significantly higher ratings on perceived usefulness and ease of use of the feedback than those in the automated feedback class. The qualitative responses from the open-ended questionnaire also reflected that, in general, the students hold more positive perceptions toward teacher feedback than toward automated feedback. One great barrier which prevents students from utilizing the comments generated by the Pigaiwang is the students’ incapability of comprehending the comments. Even though the students have positive comments on Pigaiwang’s feature of offering multiple synonyms, this does not mean that they know how to select the most appropriate word from these synonyms in the context of their writing. Students’ lack of ability to use the feedback from Pigaiwang in proper ways may have inhibited them from effectively incorporating the feedback into the revision. This may also offer some explanations as to why the revision quality in the automated feedback condition was poorer than that in the teacher feedback condition.

As no previous research has compared students’ ratings on perceived usefulness and ease of use between students receiving teacher feedback and automated feedback, it is unsure if the results found in our study represent a general pattern. It should be noted that the participants in our study are first-year university students who have just commenced their university learning. This means that our participants may lack learning autonomy due to the duck-feeding teaching style in Chinese high schools (Li and Baldauf, 2011). This may affect their perceptions of the usefulness and ease of use of the Pigaiwang as using automated feedback requires learners to have some levels of learning autonomy so that they can sustainably interact with the machine (Warschauer and Ware, 2006; Lee, 2017). Future research should be conducted with more mature Chinese EFLs, to examine their perceptions of the usefulness and ease of use of the Pigaiwang.

### Pedagogical Implications

The results of the study have some pedagogical implications for FL writing. In order to reduce teachers’ workload, college English teachers may consider using a combination of teacher and automatic feedback in FL writing classes or using the two types of feedback in rotation. As suggested by Zhang (2020), teachers should use automated feedback as “a good supplement to writing instruction and a helpful writing assistance tool in the writing and revising process” (p. 12). They may use automatic feedback to check the language errors of students’ drafts, such as spelling, punctuation, and grammar, and give students feedback on the contents and organization of their essays. Teachers should also make students fully aware of the advantages and disadvantages of automated feedback (Reynolds et al., 2021). For instance, our students commented positively about Pigaiwang’s function of providing alternative lexical items. Teachers should instruct students how to use such a function to learn vocabulary, which has been shown to be positively associated with FL learners’ writing proficiency (Staehr, 2008; Shi and Qian, 2012; Lee, 2014; Lin, 2015; Miralpeix and Muñoz, 2018).

Teachers should also consider organizing a workshop before asking students to use Pigaiwang in order for students to maximize the usefulness of the features in the Pigaiwang. In the workshop, teachers should explain all the useful functions and demonstrate the appropriate ways to use them through some concrete examples. Teachers may also need to explain different types of comments provided by Pigaiwang, such as what collocation problems are. Through this kind of workshop, students will become more prepared and more confident to navigate through the automated feedback platform, which will in turn encourage them to actively use the automated feedback during their writing and revising processes.

### Limitations and the Directions for Future Research

When interpreting the results, some limitations of the study should be kept in mind. First, our study was a relatively small-scale study, which only involved 35 participants in each feedback condition. In addition, all the participants were recruited from a single university. These limitations in sampling limit the generalizability of the study. Future research should increase the number of participants and recruit participants from different universities in order for the sample to be more representative. Second, we did not include a control group in our study, as apart from comparing students’ writing proficiency development, we also aimed to compare students’ revision quality, perceived usefulness, and ease of use of the feedback, as well as their perceptions of the strengths and weaknesses of the feedback, all of which required students to receive some forms of feedback. However, without a control group, it was difficult to rule out the possibility that students’ writing proficiency development is a result of their English learning rather than the feedback they received. The design of future studies will be significantly improved by including a control group.

Third, it should be noted that although the reliability coefficients of the perceived usefulness scale and perceived ease of use scale were all above 0.60, which was acceptable (Hair et al., 2010), they were slightly lower than the more commonly used 0.70, possibly due to the small sample size. Thus, cautions need to be taken to interpret the results related to the scales. Last but not least, while we asked the English teacher to give the feedback by covering the aspects similar to those provided in automated feedback, we did not in fact compare if the feedback provided by the teacher and Pigaiwang matched in terms of the aspects they covered. Therefore, it is unknown if the different effects of the two types of feedback on revision quality were influenced by different aspects of the two types of feedback covered. This limitation should be addressed in future research.

## Data Availability Statement

The datasets presented in this article are not readily available because of the ethics requirements of Shaanxi Xueqian Normal University. Requests to access the datasets should be directed to the Human Research Ethics Committee of Shaanxi Xueqian Normal University: kyc@snsy.edu.cn.

## Ethics Statement

The studies involving human participants were reviewed and approved by the School of Foreign Languages in Shaanxi Xueqian Normal University. Written informed consent for participation was not required for this study in accordance with the national legislation and the institutional requirements.

## Author Contributions

ZW and FH contributed substantially to the conception of the work, acquisition, analysis, interpretation of the data, drafted the work and revised it critically for important intellectual content, approved the final version of the manuscript to be published, and agreed to be accountable for all aspects of the work in ensuring that questions related to the accuracy or integrity of any part of the work are appropriately investigated and resolved. Both authors contributed to the article and approved the submitted version.

## Conflict of Interest

The authors declare that the research was conducted in the absence of any commercial or financial relationships that could be construed as a potential conflict of interest.

## Publisher’s Note

All claims expressed in this article are solely those of the authors and do not necessarily represent those of their affiliated organizations, or those of the publisher, the editors and the reviewers. Any product that may be evaluated in this article, or claim that may be made by its manufacturer, is not guaranteed or endorsed by the publisher.
